# Soluble Mediators Regulating Immunity in Early Life

**DOI:** 10.3389/fimmu.2014.00457

**Published:** 2014-09-24

**Authors:** Matthew Aaron Pettengill, Simon Daniël van Haren, Ofer Levy

**Affiliations:** ^1^Department of Medicine, Division of Infectious Diseases, Boston Children’s Hospital, Boston, MA, USA; ^2^Harvard Medical School, Boston, MA, USA

**Keywords:** plasma, serum, immunoregulatory, immune, neonatal

## Abstract

Soluble factors in blood plasma have a substantial impact on both the innate and adaptive immune responses. The complement system, antibodies, and anti-microbial proteins and peptides can directly interact with potential pathogens, protecting against systemic infection. Levels of these innate effector proteins are generally lower in neonatal circulation at term delivery than in adults, and lower still at preterm delivery. The extracellular environment also has a critical influence on immune cell maturation, activation, and effector functions, and many of the factors in plasma, including hormones, vitamins, and purines, have been shown to influence these processes for leukocytes of both the innate and adaptive immune systems. The ontogeny of plasma factors can be viewed in the context of a lower effectiveness of immune responses to infection and immunization in early life, which may be influenced by the striking neonatal deficiency of complement system proteins or enhanced neonatal production of the anti-inflammatory cytokine IL-10, among other ontogenic differences. Accordingly, we survey here a number of soluble mediators in plasma for which age-dependent differences in abundance may influence the ontogeny of immune function, particularly direct innate interaction and skewing of adaptive lymphocyte activity in response to infectious microorganisms and adjuvanted vaccines.

## Introduction

**Figure 1:**
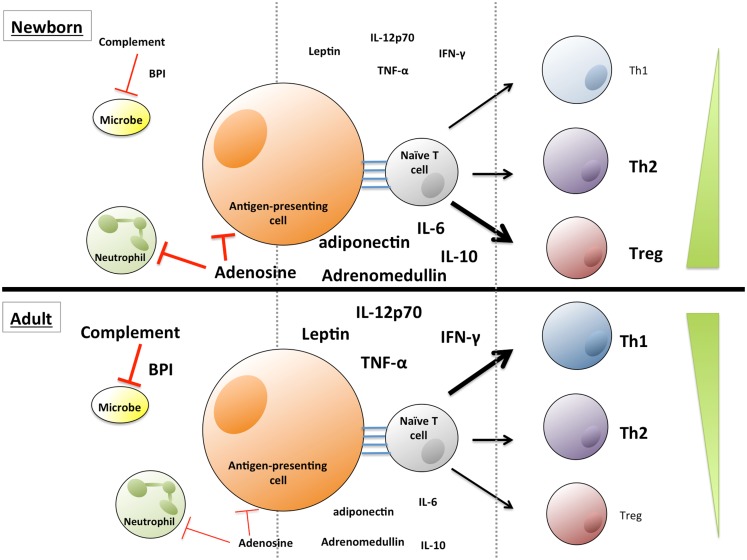
**Soluble factors influence innate and adaptive immune function and T lymphocyte polarization, and vary in concentration with age**. Lower levels of complement proteins and anti-microbial proteins and peptides contribute to neonatal susceptibility to infection, while elevated levels of adenosine, adiponectin, and adrenomedullin in neonatal blood may influence immune cell polarization. Adult blood contains lower levels of many of these immunosuppressive molecules, and adult blood leukocytes exhibit a greater propensity to produce Th1/pro-inflammatory cytokines, such as IL-12p70, TNFα, and IFNγ.

Plasma, the fluid component of blood, is a complex mixture of water, proteins, electrolytes, lipids, sugars, hormones, and gas molecules. Plasma components also infiltrate the extravascular space and tissues and have a considerable influence on many physiological processes, including being an efficient transport medium for systemic signaling. The study of plasma is complicated by the complexity of its composition – several hundred distinct proteins (1), and hundreds of small molecules (2) have been analyzed in plasma by mass spectrometry. While many of these molecules have uncharacterized functions, there is a growing evidence that many of the factors in plasma that are well-characterized help to shape the response to infection, inflammation, and immunity (3–6). Many plasma molecules vary in concentration as a function of age, and we seek here to describe both the immunoregulatory capacity of some of the best-studied molecules and the age-dependent regulation of their abundance in circulation (see Table 1) in the context of well-described deficits in neonatal immune system function (7, 8). Particular consideration is given to molecules, including cytokines, hormones, lipids, vitamins, and purines that influence the differentiation, activation, and effector functions of subsets of T cells (Figure 1). Additionally, several classes of proteins, including immunoglobulins (Igs), the complement system, and anti-microbial proteins and peptides (APPs), aid in the innate response to invading microorganisms and display age-dependent maturation (Figure 1). The critical role that plasma components play in immune function also highlights the importance of including autologous or pooled species- and age-specific plasma in the extracellular milieu in *in vitro* assay systems, instead of xenologous media (e.g., fetal calf serum), which is more commonly utilized.

**Table 1:** **Age-dependent changes in various soluble factors that influence innate and adaptive immune function, and list of references to literature regarding their concentrations in blood (Levels) and their function related to immune cell function (function)**.

Category	Molecule	Newborn/adult	Preterm/term	Refs for function	Refs for levels
Cytokines (following stimulation)	IL-6	↑	↓	(9, 10)	(11, 12)
	IL-10	↑	~	(13–16)	(3, 12, 17–20)
	IL-12p70	↓	↓	(21–23)	(11, 12, 24–28)
	IFNγ	↓	↓	(29–32)	(11, 12, 24–26, 33–35)
	TNFα	↓	↓	(29, 30)	(11, 12, 24–26)
Adipokines	Adiponectin	↑	↓	(36–38)	(39–41)
	Adrenomedullin	↑	NA	(42–45)	(46)
	Leptin	↓	↓	(47–55)	(39, 56, 57)
Complement	C1q	↓	↓	(58)	(59–61)
	C1r	↓	↓	(58)	(59, 60)
	C1s	↓	↓	(58)	(59, 60)
	C2	↓	↓	(58)	(59–61)
	C3	↓	↓	(58)	(59, 60)
	C4	↓	↓	(58)	(59–61)
	Factor B	↓	↓	(58)	(59, 60)
	Factor D	↓	↓	(58)	(59, 60)
	Properdin	↓	↓	(58)	(59, 60)
	MBL	~	↓	(58, 62–65)	(59, 60, 66–69)
	MASP	~	↓	(58)	(59, 60, 70)
	C5	↓	~	(58)	(59, 60)
	C6	↓	↓	(58)	(59, 60)
	C7	~	↓	(58)	(59, 60)
	C8	↓	↓	(58)	(59, 60, 71, 72)
	C9	↓	~	(58)	(59, 60, 71–76)
APPs	Lactoferrin	↓	↓	(77)	(78, 79)
	BPI^a^	↓	↓	(80)	(81, 82)
	Cathelicidin	↓	NA	(83)	(84)
	α-Defensins^a^	~	~	(85–87)	(81)
	β-Defensin-2	↓	↓	(4, 88)	(88)
Antibodies	IgM	↓	↓	(89, 90)	(91)
	IgA	↓	↓	(89, 90)	(91)
	IgG	~	↓	(89, 90)	(91)
Lipid-type	HDL/LDL ratio	↑	~	(92–94)	(93, 95)
Molecules	PGE2	↑	NA	(96–101)	(102)
Vitamins	Vitamin A	↓	~	(6, 103, 104)	(105)
	Vitamin D3	~	~	(106–126)	(127–130)
Purines	Adenosine	↑	NA	(131–137)	(138)

*Relative concentrations differences are shown for Newborn/Adult (↑ indicates more of soluble factor in newborn plasma/serum relative to adults, ↓ indicates less of soluble factor in newborn plasma/serum relative to adults) and Preterm/Term (↑ indicates more of soluble factor in preterm plasma/serum relative to term subjects, ↓ indicates less of soluble factor in preterm plasma/serum relative to term subjects). Levels of some soluble factors were equal, or similar, between populations (~) or were not reported in comparison between the two populations (NA)*.

*^a^BPI and α-defensins relative concentrations in neutrophil granules*.

## Cytokines

The increased susceptibility of newborns to infection is at least partially due to their impaired ability to mount a T-helper 1 (Th1) response (139). Over the last decade, several *in vitro* and *ex vivo* studies have demonstrated an impairment of neonatal leukocytes to produce Th1-polarizing cytokines, such as IL-12p70 and tumor-necrosis factor alpha (TNF-α), as compared to adult leukocytes (11, 24–26). A comparison of newborn and adult serum levels of the T-cell polarizing cytokines TNF-α and IL-6 reveals that the ratio between these cytokines during the first 7 days of life is significantly different from adults (11). TNF-α, a Th1-polarizing cytokine, is consistently low in cord blood and peripheral blood drawn during the first days of life, as compared to adult blood. In marked contrast to TNF-α, IL-6 levels in cord blood are higher than in adult blood, and continue to rise during the first days of life. IL-6 is a cytokine that is capable of inducing Th2-polarization (9) or Th17 polarization, in combination with IL-23 and TGF-β (140). In addition, it induces the production of acute-phase proteins C-reactive protein (CRP) and LPS-binding protein (LBP) (141), and has anti-inflammatory properties such as inhibition of neutrophil migration (10, 142).

In addition to distinct basal levels of serum cytokines, newborns also demonstrate a distinct pattern of cytokine production after immunization, including impairment in the production of the pro-inflammatory/Th1-polarizing cytokine IFN-γ to many vaccines (33–35), with the possible exception of bacille Calmette–Guérin (BCG) (143). IFN-γ is expressed by Th1 cells, activating macrophages to kill microbes, promoting leukocyte cytotoxicity, and inducing apoptosis of epithelial cells in the skin and mucosa (29, 30) In addition to its role in the development of a Th1 response and B-cell isotype switching (31), IFN-γ regulates MHC class I and II protein expression and antigen presentation as well (32). Overall, neonatal impairment in infection- or immunization-induced IFN-γ production is believed to be an important contributing factor in their susceptibility to intracellular pathogens. In addition, mononuclear cells from preterm newborn blood produce significantly less IFN-γ following *in vitro* stimulation than mononuclear cells from term newborns (27).

Several *in vitro* studies comparing neonatal cord and adult peripheral blood mononuclear cells have demonstrated a discordance in the secretion of T-cell polarizing cytokines after stimulation with *Toll*-Like Receptor (TLR) agonists, providing an explanation for the impairment in IFN-γ production by Th1 cells that is also observed *in vitro* (144). Whole blood assays comparing cord blood and adult peripheral blood have confirmed that newborn cells produce less TNF-α in response to common TLR agonists, such as polyinosinic:polycytidylic acid (Poly I:C, TLR3), Pam_3_CSK_4_ (TLR1/2), and lipopolysaccharide (LPS, TLR4) (138, 144, 145). Later studies supported these observations and established that newborn monocytes as well as monocyte-derived dendritic cells (MoDCs) produce less TNF-α and more IL-6 in response to these molecules (25, 28). In addition to TNF-α, newborn MoDCs also demonstrated an impairment in the production of another T-cell polarizing cytokine, IL-12p70, but appeared to be competent if not superior in the production of IL-1β (144, 146). Interestingly, newborn monocytes and MoDCs are able to produce adult-like amounts of TNF-α, Il-1β, and IL-12p70 in response to TLR7/8 agonists, such as ssRNA or the purine analog R848 (28, 146, 147). Leukocytes from preterm newborns produce less TNF-α, IL-6, and IL-12/IL-23p40 than term subjects, but similar levels of IL-10, in response to TLR stimulation (12).

IL-1β is a potent pro-inflammatory cytokine that acts as an endogenous pyrogen. It has diverse potentiating effects on cell proliferation, differentiation, and function of many innate and specific immunocompetent cells and may mediate inflammatory diseases by initiating and potentiating immune and inflammatory responses (148). IL-1β also can also act synergistically in combination with IL-6 and IL-23, enabling the expression of RORγT, which is an important step in the early development of Th17 cells (149).

Newborn leukocytes demonstrate an impaired ability to produce IL-12p70, a heterodimer that consists of a 35 kDa light chain (p35) and a 40 kDa heavy chain (p40). It is produced by activated monocytes, macrophages, neutrophils, microglia, and dendritic cells (DCs) (21). The heterodimer, IL-12p70, is a Th1-polarizing cytokine (22, 23). Production of the p35 subunit is impaired in newborn monocyte-derived DCs after treatment with LPS, correlating with a lack of nucleosome remodeling necessary for transcription factor Sp1 to gain access to the p35 promoter (150). Diminished production of the p35 subunit of IL-12p70 was also seen in newborn myeloid DCs treated with HCMV (151), and neonatal myeloid DCs produced not only less IL12p35, but also less IFN-β, as compared to adult DCs.

The molecular mechanism underlying the bias against Th1-polarizing cytokines is under active investigation. A growing literature documents that age-specific soluble plasma factors exert marked effects on TLR-mediated Th-polarizing responses (3, 25, 28, 102). Neonatal plasma contains high concentrations of adenosine (138, 152) (see Purines), an immunosuppressive metabolite that induces cyclic adenosine monophosphate in leukocytes and thereby inhibits Th1-poalrizing cytokine production. Newborn plasma enhances TLR4-mediated IL-10 production in newborn as well as adult mononuclear cells (102). This study also emphasizes that, although intrinsic cellular differences exist between newborn and adult immune cells (150, 153, 154), it is important to culture cells in autologous plasma when studying differences between age groups. In contrast, a study of neonatal mononuclear cells cultured in fetal bovine serum demonstrated impaired TLR4-mediated IL-10 production in newborn cells compared to adult cells (17). Besides macrophages and DCs, newborn regulatory B cells (Bregs) also produce IL-10 in response to TLR activation (155). Newborn cord plasma IL-10 concentrations are higher in newborns than in adults, both at baseline and after infection (3, 18–20). IL-10 inhibits the expression of co-stimulatory molecules on DCs (13), inhibits the expression of several pro-inflammatory cytokines (14) and inhibits the activation of T cells through CD28 (15). Conversely, IL-10 also enhances antibody (Ab) production by promoting B-cell survival and differentiation and increasing the production of IgG4 Abs (13). IL-10 enhances generation of regulatory T cells (Tregs) that inhibit a neonatal immune response to BCG (16, 19). How elevated levels of IL-10 affect neonatal defense against other pathogens is unclear, but is likely context dependent. Both beneficial and deleterious effects of elevated IL-10 in neonatal mice were noted upon infection with group B Streptococcus (GBS) (156, 157). On the one hand, elevated levels of IL-10 prior to GBS infection result in increased survival by reducing sepsis (156). On the other hand, elevated IL-10 levels after GBS infection can inhibit the migration of neutrophils to infected organs, resulting in increased mortality (157). Neonatal mice are impaired in their response to thymus-independent antigens, ascribed to IL-10 mediated suppression of neonatal B-cell production of IL-1β and IL-6 (20).

As a consequence of the distinct production of T-cell polarizing cytokines by newborn mononuclear cells, the adaptive immune system of newborns is skewed toward the development of Th2 and Treg cells rather than Th1 cells. Elevated levels of IL-1β and IL-6 production result in a potent acute-phase response, leading to elevated serum levels of CRP, LBP, and anti-microbial proteins and peptides (158–160), and can polarize naïve CD4 + T cells to differentiate to Th2 or Th17 cells, which can protect against bacterial or fungal infections. However, impaired production of pro-inflammatory/Th1-polarizing cytokines such as TNF-α, IFN-γ, and IL-12p70 impair the newborns’ ability to mount a protective Th1 response, leaving them vulnerable to viral infections.

## The Complement System

The complement system is a triggered-enzyme cascade of plasma proteins that deposit components with opsonin function on the surface of microbes and to membrane disruption and cell lysis on a subset of these targets (58). Complement was so named as it enhances opsonization and killing of bacteria by Ab, although it is now known that complement deposition also occurs in the absence of Ab. There are three well-defined pathways of complement activation, named for the types of molecules that trigger the cascade by binding to conserved polysaccharide patterns on the surfaces of microbes: the classical pathway, initiated by Ab binding; the mannose-binding lectin (MBL) pathway, which follows MBL recognition of distinct mannose and fucose spacing on the surface of bacteria; and the alternative pathway in which spontaneous cleavage of the complement protein C3 can lead to its deposition on the surface of microbes. These distinct early events in complement activation converge on the central event common to all three pathways – covalent attachment of the C3 convertase on the surface of the microorganism. C3 convertase cleavage of C3 generates C3b, the primary effector molecule of the complement system, and cleavage product C3a, a mediator of inflammation. C3b bound to the C3 convertase on the surface of the microorganism comprises a C5 convertase, leading to C5 cleavage and C5b attachment to the microbial surface, and the release of C5a, a peptide mediator of inflammation and potent chemokine that leads to phagocyte recruitment. C5b triggers the assembly of a membrane-attack complex that utilizes complement system proteins C6, C7, C8, and C9, to damage the membrane of susceptible bacteria. There are two primary clinical evaluations of complement function: the complement hemolysis 50% assay (CH50) in which patient serum is co-incubated with sheep erythrocytes pretreated with rabbit anti-sheep Abs and the alternative pathway hemolysis 50% assay (AP50) for which patient serum is incubated with rabbit erythrocytes in the presence of calcium chelators, which isolate the alternative pathway by inhibiting the classical and MBL pathways. Both complement function assays evaluate erythrocyte lysis mediated by dilutions of serum.

Multiple studies have characterized the ontogeny of complement expression in human plasma. CH50 is ~57–75% of adult controls for preterm subjects and 69% of adult controls for term subjects (59). AP50 values were 49, 53, and 60% of adult controls for extreme preterm (28–33 weeks GA), preterm (34–36 weeks GA), and term subjects, respectively. A review of >12 studies including preterm or term neonates, or both, shows that CH50 values for preterm subjects at GA 26–27 weeks are ~32–36% of adult controls, and that the average CH50 for term neonates, giving equal weight to each independent study, was ~59% of adult controls (60). The average AP50 for term neonates was ∼58% of adult controls, and although fewer studies evaluated AP50 in preterm neonates, the values were modestly lower than those for term neonates (60). One report showed a modest increase in CH50 activity in older adult patients (161), possibly due to increases in C4 and C9 proteins with increasing age, although not all complement components were evaluated (noted increases were gradual out to 70–79 years of age).

Levels of most individual complement proteins are lower in preterm and term neonates compared to adult levels, and while we will highlight a few examples here, the reader is referred to a recent review on the topic (60). One study demonstrated that classical and MBL pathway proteins C2 and C4 reach adult levels by 1 and 6 months of life, respectively, while classical pathway protein C1q did not reach adult levels until 18–21 months of age (61). Particularly striking among the deficiencies in levels of individual proteins are membrane-attack complex proteins C8 and C9. Levels of C8 in preterm subjects have been reported at 29% of adult levels (59, 71), and in term, subjects in the range of 36–38% of adult levels (59, 71, 72), while neonatal levels of C9 of have been more variable depending on the study, ranging from 11 to 84% of adult levels (59, 71–76). Low levels of membrane-attack complex proteins in neonates may represent a delicate balance, as C9 contributes to central nervous system and respiratory pathology related to hypoxia-ischemia (162, 163), but diminished levels of membrane-attack complex components may increase susceptibility to infection. Regarding the developmental regulation of complement proteins, it is noteworthy that while nearly all complement proteins that are found at lower levels in neonates are produced in the liver, C7, which is only modestly reduced in preterm neonates, and is at adult levels in term neonates, is not produced in the liver, but is rather largely neutrophil-derived (164).

It is unclear whether or not levels of MBL in circulation vary significantly with gestational age, with several studies demonstrating increases in MBL concentration with increasing gestational age (66–68), but a large recent study showing no such relationship (69) while still demonstrating GA dependent increases in MBL-MASP complex activity (70). What is clear is that MBL levels in plasma are highly variable due to well-characterized hereditary mutations, which effect up to 40% of the population (165, 166). Low MBL levels have been associated with increased risk of infection in adult populations (62, 63), and there is an association of MBL2 gene mutations with increased mortality and sepsis (64, 65). While one study showed no increased mortality in neonates with low MBL levels (167), several other studies have demonstrated that neonates with infections or sepsis have lower average levels of MBL or increased representation of genetic deficiency of MBL than healthy counterparts (66, 70, 168–170). Newborns may be more sensitive to genetic deficiency of MBL due to limited capacity to compensate with other pathways of complement activation. Of note, the strikingly high accumulation of genetic deficiency of MBL in humans suggests that, at least historically, there has been little evolutionary pressure on maintaining MBL activity in this population, or that this genetic locus may be subject to a variety of conflicting pressures. While this is surprising, given the strong association of low MBL levels and increased risk of infection, it may be that relatively recent changes in human health care practices are associated with the increased risk, such as biofilm formation on indwelling lines and foreign materials, or nosocomial infection, factors, which are particularly applicable to hospitalized neonatal subjects in recent decades, and to which a lower percentage of the human population was exposed in previous generations.

## Anti-Microbial Proteins and Peptides

Anti-microbial proteins and peptides play a critical role in innate immunity by directly combating susceptible pathogens and by recruiting and activating leukocytes at sites of infection. Cationic APPs that are present in blood plasma include larger proteins such as lactoferrin and bactericidal/permeability-increasing protein (BPI); peptides such as cathelicidin; α-defensins; and β-defensins.

Lactoferrin is present in mammalian secretory fluids, and also in blood plasma and neutrophil secondary granules, and has anti-microbial functions that include sequestration of iron, binding and inactivation of endotoxin, and oxidation of bacterial membrane molecules leading to membrane integrity loss (77). Lactoferrin is found at high concentrations in breast milk in particular, and may contribute to innate immune protection in early life in breast-fed children. Levels of lactoferrin are lower, however, in newborn neutrophils relative to adult neutrophils, possibly reflecting degranulation during the stress of birth (78), and plasma lactoferrin increases with increasing gestational age in preterm subjects (79). Levels of BPI are also lower in neutrophils isolated from newborns, compared to those from adult subjects (81), and lower in neutrophils from preterm newborns compared to term newborns (82). BPI is particularly active against Gram-negative bacteria and functions to neutralize endotoxin and permeabilize sensitive bacteria (80). Replenishing BPI, along with oral fluoroquinolone antibiotic, in mice rendered neutropenic by total body irradiation hastens bone marrow recovery and reduces radiation-induced mortality (171). Adjunctive recombinant BPI therapy appears to improve outcomes in children with meningococcal sepsis (172). Such studies suggest that replenishing levels of APPs in select clinical settings may be of benefit.

Human cathelicidin anti-microbial peptide 18 (hCAP-18, also called LL-37) is produced by epithelial cells, macrophages, and neutrophils, and can be upregulated in response to infection and by stimulation with the hormonally active form of vitamin D (1,25-(OH)2D3) (83). Lower serum levels of cathelicidin are associated with increased severity of acute respiratory infection in children aged 0–24 months presenting with bronchiolitis (173). Newborns have lower plasma levels of cathelicidin compared to maternal levels, with vaginal delivery associated with higher cathelicidin levels in mother and newborn compared to caesarian section (84).

α-Defensins and β-defensins are cationic peptides produced by a wide variety of organisms with anti-infective activity against viruses (85), bacteria (86), and fungi (87). There are 6 α-defensins, four of which are predominately produced by neutrophils (human neutrophil peptides 1-4, HNP1-4), which are expressed at adult-like levels at birth (81). β-Defensins are produced primarily by epithelial cells, macrophages, and neutrophils, and in addition to direct anti-microbial activity related to microbial membrane disruption, also function as chemotactic peptides to recruit particular classes of leukocytes (4). Low serum levels of β-defensin-2 have been associated with increased risk of developing sepsis in preterm neonates (88). This study also noted that β-defensin-2 is higher in term than in preterm serum, and that levels of β-defensin-2 correlated with gestational age and weight. Overall, APPs likely play an important role in fetal and neonatal innate immunity, helping to regulate colonization and resisting infection (174).

## Antibodies

The composition of Ig isotypes in newborns and infants is distinct from that of adults, with IgG, initially primarily of maternal origin, near adult levels but rapidly declining to a nadir of circulating IgG at about 3 months of age, and significantly reduced levels of IgA and IgM at birth that gradually rise to near adult levels by puberty (91). Fetal B cells begin producing small amounts of Ig in the 20th week of gestation, predominantly IgM antibodies (Abs), with limited VH-gene segment usage (175, 176). The majority of circulating Abs in newborns is, however, of maternal origin. Maternal Igs, which are transported across the placenta during pregnancy, contribute to the protection of infants from infectious diseases during the first months of life. In addition to their role in binding antigen (89), Igs also play an important role in regulating adaptive immune responses through their interaction with Fc receptors (FcRs) (90). As a result, the presence of Igs during the first months of life can influence how newborns and infants respond to vaccination.

Maternal antibodies (MatAbs) are transported across the feto-maternal interface with the help of receptors that are specific for the Fc-portion of IgG: FcRn and FcγR I, II, and III (177). Accordingly, only Igs of the IgG isotype are transported across the placenta. Of the different IgG subclasses, IgG1 is the most efficiently transported subclass and IgG2 is the least (178). In general, IgG is the most potent of all isotypes with respect to Fc-receptor binding on macrophages and NK cells. It is also able to activate complement, though less potently than IgM.

As MatAbs are a crucial component of the humoral immune system of newborns, it is important to note that the effector function of these Abs changes dramatically during pregnancy. The Th2-polarized cytokine environment during pregnancy drives an essential change in the glycosylation pattern of the mothers’ IgG, resulting increasing asymmetrically glycosylated IgG Abs (179). These Abs possess a mannose-rich oligosaccharide residue bound to one of the Fab regions, making them unable to activate immunoeffector mechanisms, such as complement fixation, and clearance of antigens and phagocytosis (179). Because the glycosylation does not affect binding to FcRn, asymmetrically glycosylated Abs can be found in the fetal circulation as well (180). These asymmetrical MatAbs can of course still confer protection by binding to pathogenic antigens.

The protective effects of MatAbs on the newborn depend on the gestational age of the fetus at birth. Premature infants are often vulnerable to infections, partly because of the low transplacental transfer of MatAbs. For example, transplacental transfer of MatAbs against varicella zoster virus (VZV) is significantly lower in preterm infants born at ≤28 weeks gestational age, compared with those in preterm infants 29–35 weeks gestational age and term infants (181). Similarly, protective, neutralizing Abs specific for Rubella, or cytomegalovirus (CMV) are present at higher concentrations in the circulation of full-term infants, as compared to preterm infants, contributing to preterm susceptibility to these viruses (182, 183).

As MatAbs may contribute to protection against infections during the first 6 months of life, maternal immunization has been a strategy of interest. Indeed, this approach has proved safe and beneficial to immunize mothers, such as in the case of Tdap, but this approach may reduce the infants’ response to their own primary Tdap immunization at 6 months (184). Inhibition of vaccine efficacy by MatAbs is particularly evident with live viral vaccines such as measles or respiratory syncytial virus (RSV) (185, 186), but has also been observed with a conjugate vaccine against *Neisseria meningitidis* (187). Proposed mechanisms of inhibition are epitope masking and B-cell inhibition by cross-linking of the B-cell receptor with FcγRIIB.

## Hormones

Hormones regulate physiological functions of many cells types including leukocytes. Two critical hormones that circulate in plasma and influence immune cell function are leptin and adiponectin, known as “adipokines” – cytokines produced primarily by adipose tissue. Leptin is a 16 kDa protein hormone that regulates hunger/satiety sensation and metabolic rate, and is produced in relation to the mass of adipose tissue. Leptin concentrations fluctuate considerably based in part on satiety, which increases leptin, or starvation, which decreases leptin. Adiponectin is a ~30 kDa metabolic regulatory protein hormone that modulates glucose levels and fatty acid oxidation (188). Intriguingly, stimulation of the leptin receptor activates signal transducer and activator of transcription 3 (STAT3), STAT5, and Janus kinase 2 (JAK2) inducing gene transcription via IL-6-responsive gene elements (189). Adiponectin shares sequence homology with a complement system protein (C1q) and structural homology with TNF family members (190). The full-length LepRb leptin receptor is expressed in T cells, NK cells, macrophages, and polymorphonuclear cells (5). Genetic deficiency of leptin in human beings has been associated with reduced CD4 T-cell populations, hyporesponsive T cells, and lower levels of IFNγ, while increasing levels of transforming growth factor β (TGFβ), conditions which were reversed with leptin replacement therapy (47). Similar immunological dysfunction has been noted in subjects with leptin receptor deficiency (48, 49). In mice, leptin protects against infection with *Mycobacterium tuberculosis* (50), *Klebsiella pneumonia* (51), and *Streptococcus pneumoniae* (52–54). *In vitro* stimulation of cord blood and adult T cells with leptin leads to upregulation of IFNγ, IL-2, IL-4, and IL-10, and interestingly significantly more IFNγ was produced by female cord blood T cells than from male (55). Circulating leptin levels at birth are lower than, and influenced by, maternal levels of leptin (39) and are higher in term neonates than in preterm neonates (56). Leptin levels drop in the first days of life (39, 56) and then gradually rise with age peaking at puberty (57). There is moderate sex dimorphism in serum leptin levels – females having significantly higher levels of leptin in older age groups (56, 57).

Adiponectin is found at very high levels in serum, in the range of ~10 μg/ml (40), is elevated at birth compared to maternal levels (41), and does not drop in the first 4 days of life (39). Circulating adiponectin levels are lower in preterm newborns than term newborns (191), and levels decrease from term birth to approximately adult levels by 6–10 years of age (40). Adiponectin induces anti-inflammatory IL-10 production from human monocytes, macrophages, and DCs, while suppressing TNFα production (36). Adiponectin receptors are upregulated on T cells following activation, and adiponectin stimulation of CD8 + T cells inhibits proliferation and IL-2 production, as well as production of the pro-inflammatory cytokines IFN-γ and TNFα (37). Additionally, adiponectin suppresses DC IL-12p40 production and co-stimulatory molecule expression, and in DC/T-cell co-cultures favor the generation of regulatory T cells (38).

Adrenomedullin (AM), a cleavage product of preproadrenomedullin, is a circulatory neuropeptide hormone. AM stimulates calcitonin receptor-like receptor (CALCRL) or receptor activity-modifying proteins 2 and 3 (RAMP2 and RAMP3), leading to increased cyclic-AMP (42). AM is generated by a variety of leukocytes in response to inflammatory stimuli (43), and also has direct anti-microbial activity (44). Pretreatment of mice, or murine macrophages, with AM before challenge with endotoxin suppressed the production of pro-inflammatory cytokines (e.g., TNFα, IL-1β, CCL5, IL-12) while promoting IL-10 production, and reduced mortality due to cecal-ligation and puncture-induced sepsis (45). Plasma levels of AM are elevated in pregnant mothers and in cord blood relative to non-pregnant female levels (46), which could potentially influence the suppression of pro-inflammatory cytokine production that has been noted in both neonates and late in pregnancy for mothers.

Sex hormones (estrogen, progesterone, and testosterone) can also influence the immune system (192–194), which may contribute to gender differences in responses to infection and immunization (195–197), but they have primarily been studied in this context in animal studies utilizing genetically modified organisms with a complete lack of various enzymes or receptors involved in steroid production or signaling. Androgens (including testosterone) generally suppress immune cell function *in vitro*, while estrogen may enhance Ab production (198). Estrogen (estradiol) in females and testosterone in males are found at relatively low levels at birth, increase after puberty reaching their highest levels during teenage years (10, 33, 34, 141, 142), and diminish thereafter (57), but it is unknown if lower levels of these hormones at birth influences immune cell activity. Increased levels of progesterone during pregnancy may modulate the Th1/Th2 profile of adaptive responses – favoring production IL-4 and IL-5 and Th2 bias (199, 200). Progesterone levels are elevated at birth (presumably of maternal origin) in both genders relative to later in childhood, but not to levels found in female adults (201).

## Lipids and Lipid-Type Molecules

Pregnancy affects maternal metabolism of various substrates and nutrients including lipids, which affects innate immunity. Changes in the maternal plasma lipid profile include increased concentrations of fatty acids, triglycerides, and cholesterol and by changes in the concentration and composition of lipoproteins (202–204). Newborns have lower levels of total cholesterol, with a preponderance of high-density lipoprotein (HDL), as opposed to the abundance of low-density lipoprotein (LDL) in adult blood (92–94). Moreover, the composition of fetal HDL particles is distinct from that of adults. Fetal HDL particles are enriched in apolipoprotein E (apoE) and have diminished levels of apoA-1 and apoL, as compared to maternal HDL (93). The lipoprotein composition of fetal HDL can vary between male and female newborns as well, as female newborns have higher levels of HDL-cholesterol than male newborns (205, 206). Distinct fetal HDL composition affects fetal endothelial cell function and tissue growth (94), as well as the developing immune system. In general, the elevated ratio of HDL/LDL in newborns is associated with immune suppression (93). Preterm newborns have elevated levels of cholesterol, but similar HDL/LDL ratios compared to term newborns (95). HDL is an acute-phase reactant that can bind and neutralize LPS. IL-6 can alter the composition of HDL particles, resulting in less apoA-1 expression and Serum paraoxonase/arylesterase 1 (PON1) activity, in turn reducing its anti-oxidant properties (207). Given HDL’s roles in clearance of endotoxin (208), reduced levels of apoA-1 in newborns may affect their susceptibility to sepsis. Most other immune-modulating activities of HDL, such as down-regulation of co-stimulatory molecules on macrophages and DCs (209) and TNF-α-induced expression of adhesion molecules on endothelial cells (210) have been largely ascribed to the presence of apoA-1. Despite the apparent reduction of apoA-1 in neonatal HDL particles, newborn HDL is also immunosuppressive via the activity of apoE, which has the ability to inhibit T-cell proliferation and nitric oxide synthesis by macrophages (211, 212).

Another important lipid mediator of the newborn immune system is prostaglandin E_2_ (PGE_2_). PGE_2_ is a prostanoid that is generated from arachidonic acid by the action of cyclooxygenase isoenzymes. It can function in both the promotion and the resolution of inflammation. PGE2 signals via G-protein coupled Prostaglandin E receptors expressed on a variety of immune cells, including DCs and T cells (96, 97). PGE_2_ is elevated in newborn plasma, as compared to adults (102). Although PGE_2_ inhibits IL-12p70 production (98), it is not solely responsible for impaired TLR4-mediated IL-12p70 production in newborns as additional yet to be identified soluble plasma components appear to contribute to that activity (102). The pleiotropic nature of PGE_2_ precludes a simple analysis of its overall affect on the newborn immune system. In general, PGE_2_ inhibits Th1-polarizing cytokine production by DCs and macrophages, changes DC morphology, resulting in a loss of podosome formation and co-stimulatory receptor expression (99–101). Paradoxically, PGE_2_ may also increase production of Th1-polarizing cytokines and DC function (213, 214). These apparently conflicting *in vitro* activities may be due to distinct effects that PGE_2_ exerts over time and at different DC:T cell ratios in co-culture (215, 216), as PGE2 can also act directly on CD4 + T cells, promoting the expansion of Th1 and Th17 cells (217). Stimulus-induced production of PGE_2_ by a human mono-mac cell line *in vitro* may correlate with the tendency of vaccine adjuvants to induce fever *in vivo* (218). Overall, it is likely that elevated levels of PGE_2_ contribute to the acute-phase response as well as to the skewed polarization of T-helper cells in newborns.

## Vitamins

Vitamins, especially -A and -D, exert considerable influence on both innate and adaptive immune cell function (6, 103). Vitamin A enhances T-cell proliferation, likely by increasing IL-2 production (104) as well as DC maturation, antigen presentation, and migration (219). Vitamin A-deficient mice exhibit defects in helper T-cell activity (220). Serum vitamin A levels are influenced by diet and supplementation, but apparently only moderately by age (105).

Vitamin D3 is generated in the skin on exposure to sunlight or acquired in the diet from animal sources, fish in particular, whereas vitamin D2 is derived from plants. Both are utilized in supplementation, although vitamin D3 metabolites have higher affinities at human vitamin D binding proteins and receptors than vitamin D2 metabolites and therefore vitamin D3 may be considered preferable due to higher bioefficacy (221). Vitamin D3 suppresses lymphocyte function *in vitro*. The active vitamin D3 metabolite 1,25-dihydroxyvitamin D3 (1,25-(OH)2D3) inhibits T-cell proliferation (106, 107), production of IL-2 (107–109), and the Th1-polarizing cytokine interferon-γ (IFNγ) (110, 111), while increasing production of the Th2 cytokine IL-4 (112). These effects are more pronounced in the effector T-cell subset that exhibits high expression of the vitamin D receptor (VDR) (113). Additionally, 1,25-(OH)2D3 impacts the capacity to activate Th1 T-cell responses by suppressing DC maturation and DC production of Th1-polarizing cytokine IL-12 (both the p35 and p40 subunits, thus preventing IL-12p70 and IL-12p40 assembly) but increasing the production of IL-10 (114, 115) which favors Treg differentiation (116, 117). The 1,25-(OH)2D3 also inhibits B-cell effector functions (106, 118), likely via 1,25-(OH)2D3 suppression of antigen-presenting cell function (119). The 1,25-(OH)2D3 stimulation of monocytes and macrophages, however, increases proliferation (120) and cathelicidin anti-microbial peptide production (108, 121), and activates the cellular process of autophagy (122), which can destroy intracellular bacteria, such as *M. tuberculosis*. Accordingly, vitamin D deficiency has been associated with increased risk of tuberculosis in several populations (123, 124), including children (125, 126). Circulating concentrations of vitamin D are heavily influenced by factors such as diet, supplementation, socioeconomic status, and season (127). Several studies have assessed the ontogeny of serum vitamin D levels. Cord serum levels of 1,25-dihydroxyvitamin D [1,25-(OH)2D] are moderately reduced but reach adult levels in neonatal peripheral blood by 24 h of age (formula feeding considered not likely to be the source of 1,25-(OH)2D in this study) (128). There are moderate increases in 1,25-(OH)2D during puberty in both sexes (129). In healthy subjects, 20–94 years of age neither serum 25-hydroxy- nor 1,25-dihydroxyvitamin D [25OHD and 1,25-(OH)2D] changes with age in either sex (130). While it is unclear what the impact of age is on circulating levels of vitamin D, it is clear that vitamin D levels play a critical role in neonatal and infant health, and that acquisition of vitamin D in these populations is amenable to supplementation and dietary modification (222). Vitamin D deficiency certainly needs to be combated, but it may also be true that supplementation could influence immune function by polarizing the adaptive immune response toward a Th2 profile, which should be a topic for future research.

Other vitamins and minerals, such as vitamins C and E, B vitamins, and trace elements, can also impact immune function (223) and warrant consideration in populations with limited dietary access to these molecules.

## Purines

Plasma purine nucleotides and nucleosides, particularly adenosine triphosphate (ATP), adenosine diphosphate (ADP), and adenosine, are critical signaling molecules that regulate the function of a wide variety of cells, including immune cells. Extracellular ATP (eATP) influences T-cell activation (224, 225) and proliferation (226, 227), promotes neutrophil/endothelial cell adhesion (228), degranulation (229, 230), and reactive oxygen species (ROS) production (231), as well as other pro-inflammatory immune cell functions (232). eATP is transported to the extracellular space by vesicular trafficking, secreted via pannexin-1 channels (233), or released in large quantities from necrotic cells. Extracellular ADP (eADP) can be derived from eATP via dephosphorylation, and is also released into circulation from platelets following activation. eATP and eADP can stimulate P2 purinergic receptors and also serve as a source of adenosine (Ado) through dephosphorylation by several types of ecto-nucleotidases leading to adenosine receptor signaling (234). Extracellular adenosine (eAdo) has a nearly opposite profile of immune cell regulating effects from the precursor ATP: eAdo inhibits neutrophil-endothelial adhesion (131, 132) and effector functions (133–135), macrophage production of pro-inflammatory or Th1-polarizing cytokines (IL-12p70, TNF-α) (232), and T-cell proliferation and effector functions (136, 137). By modulating the amount of adenosine, enzymes that metabolize extracellular purines, including several families of ecto-nucleotidases (235) and adenosine deaminase (ADA1), help regulate whether signaling is tilted toward a P2 receptor-mediated pro-inflammatory response, or a P1 receptor-mediated anti-inflammatory response. Cord blood plasma contains significantly higher levels of adenosine than adult peripheral blood plasma (138). In addition, the purine enzyme profile in cord blood plasma – elevated AMP dephosphorylating activity (alkaline phosphatase and soluble CD73) but lower adenosine deaminase activity compared to adults – favors generation of adenosine from purine nucleotides (152).

## Conclusion

Blood plasma contains a complex mixture of bioactive molecules, including proteins, sugars, hormones, vitamins, and purines, many of which have influence on the host response to infection. The distinct molecular composition of blood plasma at birth and during the neonatal period contributes to distinct immunological function in newborns. A molecular milieu that blunts pro-inflammatory/Th1-polarizing responses likely serves to protect *in utero* against maternal adaptive non-self immune responses, and may help mediate the transition to the foreign antigen-rich *ex utero* environment. However, this polarization may come at a cost with respect to impaired host defense against intracellular pathogens. Such immune polarization and heightened susceptibility are especially evident in preterm neonates that have lower levels of maternal antibodies and certain complement proteins as compared to term subjects, leaving them particularly dependent on endogenous defense against infection. Of note, immunization of neonates with BCG modulates Ab production to both HBV and oral polio vaccination (OPV) (236), which, given the different routes of administration, suggests a role for soluble mediators induced by one vaccine impacting on the subsequent immune response to another. Cautious manipulation of the immunoregulatory capacity of neonatal blood plasma by targeting specific molecules and signaling pathways may optimize responses to infection and immunization.

## Conflict of Interest Statement

The authors declare that the research was conducted in the absence of any commercial or financial relationships that could be construed as a potential conflict of interest.
